# 继发于系统性红斑狼疮的复发免疫性血栓性血小板减少性紫癜1例报告并文献复习

**DOI:** 10.3760/cma.j.cn121090-20241128-00489

**Published:** 2024-12

**Authors:** 竑 田, 云 李, 海菊 何, 晓燕 徐, 自强 余, 杰 殷

**Affiliations:** 国家血液系统疾病临床医学研究中心，江苏省血液研究所，苏州大学附属第一医院，国家卫生健康委员会血栓与止血重点实验室，苏州 215006 National Clinical Research Center for Hematologic Diseases, Jiangsu Institute of Hematology, the First Affiliated Hospital of Soochow University, Key Laboratory of Thrombosis and Hemostasis of Ministry of Health, Suzhou 215006, China

**Keywords:** 免疫性血栓性血小板减少性紫癜, 复发, 系统性红斑狼疮, 硼替佐米, Immune thrombotic thrombocytopenic purpura, Relapse, Systemic lupus erythematosus, Bortezomib

## Abstract

**目的:**

报道1例继发于系统性红斑狼疮（SLE）的复发免疫性血栓性血小板减少性紫癜（iTTP）患者的诊疗过程，并通过复习相关文献，为该疾病的诊治提供参考依据。

**方法:**

回顾性分析苏州大学附属第一医院收治的1例继发于SLE的复发iTTP患者的诊疗过程，并进行相关文献复习。

**结果:**

该患者iTTP复发的同时合并了狼疮中度活动、低纤维蛋白原血症、肾功能不全、肺部感染。在采用血浆置换（TPE）、激素冲击、硼替佐米以及多学科诊疗团队综合诊治后，患者的iTTP及SLE均获得缓解。

**结论:**

诊断iTTP的同时须完善全面检查，以排除合并其他自身免疫性疾病的可能。对于复发、难治的病例可尝试如硼替佐米等针对浆细胞的治疗。

## Abstract (en)

**Objective:**

To review the diagnosis and treatment process of a patient with relapsed immune thrombotic thrombocytopenic purpura（iTTP）secondary to systemic lupus erythematosus（SLE）and conduct a review of relevant literature, so as to provide a reference basis for the diagnosis and treatment of this disease.

**Methods:**

We retrospectively analyzed the diagnosis and treatment process of a patient with relapsed iTTP secondary to SLE who was admitted to the First Affiliated Hospital of Soochow University, and conducted a review of literature.

**Results:**

When the patient's iTTP relapsed, it was accompanied by moderate lupus activity, hypofibrinogenemia, renal insufficiency, and pulmonary infection. After adopting therapeutic plasma exchange（TPE）, hormone pulse therapy, bortezomib, and comprehensive diagnosis and treatment by a multidisciplinary team, both the patient's iTTP and SLE were relieved.

**Conclusion:**

A comprehensive examination should be completed when diagnosing iTTP to rule out the possibility of combining with other autoimmune diseases. For relapsed and refractory cases, treatments targeting plasma cells, such as bortezomib, can be attempted.

免疫性血栓性血小板减少性紫癜（iTTP）是一种罕见且严重的血栓性微血管病（TMA），系血管性血友病因子裂解酶（ADAMTS13）抑制物所致[1]。部分iTTP继发于自身免疫性疾病[2]。iTTP合并系统性红斑狼疮（SLE）发生率仅为0.5％～2％，但死亡率高达34.1％～62.5％[3]–[5]。治疗性血浆置换（TPE）、激素、利妥昔单抗是iTTP一线治疗方案[6]，然而部分患者仍会出现治疗无效或复发。现将我院收治的1例继发于SLE的复发iTTP患者的诊疗过程报道如下，并复习相关文献。

## 病例资料

患者，女，27岁。于2022年1月因“四肢瘀点、发热、头晕呕吐、尿色加深”首次至我院就诊，门诊查血常规：WBC 3.9×10^9^/L，HGB 63 g/L，PLT 6×10^9^/L；生化：总胆红素31.2 µmol/L，肌酐186 µmol/L，乳酸脱氢酶1705 U/L；溶血组套：血浆游离血红蛋白阳性，遂收住入院。入院后查外周血破碎红细胞10.5％；抗核抗体谱：抗ANA阳性（+），抗Histones阳性（+），抗U1-snRNP阳性（+）；24 h尿蛋白：4.38 g；头颅CT未及异常；TTP组套：血浆ADAMTS13活性0％，ADAMTS13抑制物阳性。遂诊断为iTTP、未分化结缔组织病、肾功能不全。予甲泼尼龙、8次TPE后，患者的PLT回升至148×10^9^/L，肌酐降至90.3 mmol/L，乳酸脱氢酶降至364.3 U/L。同时给予利妥昔单抗［700 mg（375 mg/m^2^），每周1次，共用2次］。但患者未遵嘱完成后续2次利妥昔单抗的治疗，亦未规律复诊。

2022年5月患者因“四肢瘀斑、面部红斑，月经增多两周”再次就诊，门诊复查HGB 61 g/L，PLT 16×10^9^/L。入院查体：神志清，精神软，体型肥胖（身高164 cm，体重92 kg），贫血貌，面部可见蝶形色素沉着斑，双手指腹可见红色不规则形状红色皮疹，压之不褪色，双下肢散在瘀斑，双下肺呼吸音减低，未及啰音，心脏及腹部查体无特殊，双下肢非凹陷性水肿。完善检查，血常规：WBC 5.44×10^9^/L，HGB 61 g/L，PLT 16×10^9^/L，网织红细胞3.1％，破碎红细胞3％；TTP组套：血浆ADAMTS13活性0％，ADAMTS13抑制物阳性；生化：间接胆红素21.9 µmol/L，白蛋白24 g/L，乳酸脱氢酶603.3 U/L，肌酐259 µmol/L；尿常规：尿蛋白（+++）；血凝常规：APTT 27.1 s，PT 13.6 s，TT 20.5 s，纤维蛋白原（FIB）1.02 g/L，ATⅢ 92％, D-二聚体0.79 mg/L，FDP 2.63 mg/L；胸痛组套：高敏肌钙蛋白T 48.59 pg/ml，心房钠尿肽3227 pg/ml；抗核抗体谱：抗ANA（+）颗粒型，抗ds-DNA阳性（+），抗Histones阳性（+），抗U1-snRNP阳性（+）；抗dsDNA 10.2 IU/ml；补体4 0.04 g/L，补体3 0.30 g/L；胸部CT：少许肺部感染。

患者确诊iTTP（复发）、SLE（SLEDAI评分14分）、肾功能不全、肺部感染，经5次TPE、常规剂量糖皮质激素（甲泼尼龙80 mg/d）治疗后血小板逐步回升。由于患者系SLE继发的iTTP，且利妥昔单抗治疗后短期复发，遂予硼替佐米［2.6 mg（1.3 mg/m^2^）每周两次，连用2周］治疗，同时予N-乙酰半胱氨酸、抗感染、补充白蛋白、利尿等支持治疗。患者本次入院时查FIB降至1.02 g/L，补充FIB（2 g/d，间断使用2次）后可回升至正常水平（3.41 g/L），但停止补充后逐步下降至入院时水平。

尽管予激素冲击（甲泼尼龙500 mg/d，连用3 d）、硫酸羟氯喹控制SLE活动，间断补充人血白蛋白营养支持，同时限制水摄入并加强利尿，患者的体重仍上涨了14 kg，腹部皮肤多发张力性水疱，肌酐亦升至280 µmol/L，同时还存在高钾血症、高磷血症、低钙血症。血液科组织了心内科、风湿科、肾内科、重症、营养科等开展多学科诊疗（MDT），并将患者转入血液ICU进一步诊治。经床边连续性肾脏替代治疗减轻容量负荷，激素、环磷酰胺（0.6 g/次，每周用2次）、硫酸羟氯喹控制狼疮活动，继续间断血浆置换、硼替佐米治疗iTTP，同时预防血栓、营养支持、维持电解质平衡、保肾、预防感染等综合治疗后，患者体重进行性下降，尿量逐步增加至每日1000 ml，PLT维持在100×10^9^/L以上。随着SLE及TTP病情得到控制，该患者FIB水平也趋于正常。随后，该患者接受了第二疗程的硼替佐米巩固治疗。患者至今规律服用吗替麦考酚酸酯、硫酸羟氯喹等控制狼疮，随访27个月，TTP及SLE均处于缓解状态。

## 讨论及文献复习

TTP是一种罕见的临床急重症，其特征为严重的血小板减少、微血管病性溶血性贫血以及终末器官功能障碍。该病十分凶险，若未及时行TPE，死亡率高达90％[7]。iTTP的发生多无明确原因，但也可能继发于感染、药物、肿瘤、自身免疫性疾病、造血干细胞移植等。我们对2017年至2022年间在本中心诊治的78例iTTP进行了回顾性分析，约30％的患者合并自身免疫性疾病，这与以往的研究报道相类似[8]，其中SLE发生率约占这些自身免疫性疾病的20％，存在SLE基础的iTTP患者病死率显著升高。既往的经验提示，治疗iTTP的免疫抑制剂也有助于控制其他的自身免疫性疾病[9]。因此，确诊iTTP的同时需完善相关检查，以排查潜在的其他自身免疫性疾病。

利妥昔单抗能够降低早期死亡率、减少复发、延长缓解，显著改善iTTP的预后，已被写入指南[6]。但长期随访数据表明，443例iTTP患者随访5年的复发率达40％[10]。然而定期监测ADAMTS13并预防性利妥昔单抗治疗，能显著降低复发率（22.6％对11.1％，*P*＝0.0004）[10]。虽然利妥昔单抗疗效确切且不良反应较小得到了临床认可，但是仍有一部分患者在接受含TPE、激素、利妥昔单抗的标准治疗后无法实现ADAMTS13持续缓解或复发[11]，因此对利妥昔单抗耐药/复发的iTTP患者的治疗成为了一项亟待解决的临床难题。

表1总结了十余年来对利妥昔单抗治疗耐药或复发的iTTP治疗方案的文献报道，涵盖了靶向作用于B淋巴细胞的奥妥珠单抗、奥法木单抗，以及针对浆细胞的硼替佐米和CD38单抗。部分患者无法耐受利妥昔单抗输注反应或者在采用利妥昔单抗治疗后产生抗利妥昔单抗抗体，近年来上市的奥妥珠单抗、奥法木单抗等人源化抗CD20单抗克服了这些问题，因而被用于无法耐受利妥昔单抗输注反应或利妥昔单抗耐药/复发的患者，已取得了一定的效果[12]–[13]。目前的主流观点认为，部分患者利妥昔单抗治疗无效或复发的原因可能在于他们体内存在长寿浆细胞[24]，这群细胞的CD20常表达缺如，但CD38呈阳性。蛋白酶体抑制剂硼替佐米在多发性骨髓瘤的治疗中已被广泛应用，它对自身反应性的短寿浆细胞和长寿浆细胞具有促凋亡作用[25]。2013年首次报道了硼替佐米用于难治性iTTP的治疗[14]，该患者对激素、环磷酰胺、利妥昔单抗等免疫抑制剂治疗均无反应，但在硼替佐米治疗后，患者脱离了血浆依赖，获得缓解。此后，陆续有将硼替佐米用于iTTP的个案报道[21]。硼替佐米治疗iTTP的机制尚不完全清楚，但可能与清除残留的反应性B细胞和浆细胞，以及抑制树突状细胞将ADAMTS13抗原向CD4^+^ T细胞递呈，从而减少ADAMTS13抑制物产生有关。本中心曾总结了将硼替佐米应用于对利妥昔单抗耐药或复发的iTTP，研究中所有复发患者均获得了持续的临床缓解[20]。在狼疮小鼠模型中，硼替佐米也已被证实可减少浆细胞数量、改善狼疮性肾炎并延长生存期[26]。随后在部分自身免疫性疾病（包括SLE）中也证实，硼替佐米可通过清除产生自身抗体的长寿浆细胞来治疗疾病[27]–[28]。本例病例再次证实了硼替佐米在有狼疮背景的利妥昔单抗复发iTTP患者中的有效性。

**表1:** 部分现有报道的利妥昔单抗后耐药/复发免疫性血栓性血小板减少性紫癜治疗方案及效果

文献来源（患者例数，出版年份）	性别	年龄（岁）	平均复发次数	本次治疗前应用过的方案	复发/难治状态下采用治疗（用法）
Ahmadpoor等[12]（1例，2021）	女	19	2	TPE，CS，RTX，CAP	OTZ（1000 mg第0，15，21天）
Doyle等[13]（15例，2022）	–	15	–	TPE，CS，RTX，CSA/CTX/MMF	OTZ（总量2000～3000 mg） OFM（总量1000～4000 mg）
Shortt等[14]（1例，2013）	女	53	0	TPE，CS，CTX，RTX，NAC	Bz（1.3 mg/m^2^ 第1, 4, 8, 11天，共4个疗程）
Yates等[15]（1例，2014）	女	48	8	TPE，RTX，CTX	Bz（1.3 mg/m^2^第1, 4, 8, 11天，共13次）
Patel等[16]（1例，2016）	女	22	0	TPE，CS，RTX	Bz（1.3 mg/m^2^第1, 4, 8, 11天，共2个疗程）
Patriquin等[17]（6例，2016）	3男 3女	27～76	0	TPE，CS，RTX	Bz（1 mg·m^−2^·周^−1^，每例给药1～3次）
Baseri等[18]（1例，2019）	女	44	0	TPE（以白蛋白溶液作为替代液）, CS, RTX, NAC,血管性血友病因子Ⅷ浓缩物	Bz（1.3 mg/m^2^，共4次）
Maschan等[19]（2例，2021）	女	5～12	0	TPE，CS，IVIG，RTX；TPE，CS，RTX	Bz（1.3 mg/m^2^第1, 4, 8, 11天，共6次）
Yin等[20]（4例，2022）	3男 1女	18～46	4	TPE，CS，RTX（2例）; TPE，CS，RTX，CSA（1例）; TPE，CS，RTX，CSA，NAC（1例）	Bz（1.3 mg·m^−2^·周^−1^，共8次）
Lee等[21]（8例，2024）	4男 4女	30～67	4（1-8）	TPE，CS，RTX（3例）; TPE，CS，CTX，RTX（1例）; TPE，CS，RTX，CAP（3例8）;TPE，CS，RTX，CSA（1例）	Bz（1.3 mg·m^−2^·周^−1^，共4～13次）; Bz（1 mg·m^−2^·周^−1^，共4次）
van den Berg等[22]（2例，2022）	1男 1女	31，32	0，2	TPE，CS，CAP，RTX	Dara（16 mg·kg^−1^·周^−1^×4周）
Weisinger等[23]（7例，2024）	3男 4女	39～70	8次ADAMTS13复发，1次临床复发	TPE，CS + RTX（7例）/CSA（3例）/OFM（2例）/Bz（1例）	Dara（16 mg·kg^−1^·周^−1^×4～10周，此后有2例患者每月1次维持治疗）

**Table:** 

文献来源（患者例数，出版年份）	疾病状态	达到ADAMTS13缓解（活性≥20％）的天数	不良反应	随访时间（d）	末次随访时的状态
Ahmadpoor等[12]（1例，2021）	复发/难治（抗利妥昔单抗抗体）	–	无	120	CR
Doyle等[13]（15例，2022）	复发（3例存在抗利妥昔单抗抗体）	达ADAMTS113活性峰值的中位时间为120（66～155）d	2例需要治疗的感染，2例1级输注反应	252（198～426）	无复发生存时间522（447～1380）d
Shortt等[14]（1例, 2013）	难治	29 d脱离TPE	无	4	临床反应，ADAMTS13部分缓解
Yates等[15]（1例，2014）	复发/难治	44	无	169	CR
Patel等[16]（1例，2016）	难治	106	神经病变	540	CR
Patriquin等[17]（6例，2016）	难治	出院时6例患者ADAMTS13活性：87％，89％，死亡，83％，83％，75％	无	90～990	CR
Baseri等[18]（1例，2019）	难治	–	无	210	临床反应
Maschan等[19]（2例，2021）	难治	–	无	360～1800	CR
Yin等[20]（4例，2022）	复发/难治	–	血小板减少（2例），中性粒细胞减少（2例）	180～1170	CR
Lee等[21]（8例，2024）	复发/难治	5例CR：17～243；2例NR；1例21 d达PR	1例肺功能下降	120～2670	3例维持CR；2例NR；2例ADAMTS13复发；1例临床复发
van den Berg等[22]（2例，2022）	临床反应但ADAMTS13未缓解	7	无	77～274	CR
Weisinger等[23]（7例，2024）	复发/难治	28	5例Dara相关输注不良反应，无严重不良反应	360～1470	12个月无复发生存率56％

**注** TPE：血浆置换；CS：激素；CTX：环磷酰胺；RTX：利妥昔单抗；NAC：N乙酰半胱氨酸；OTZ：奥妥珠单抗；OFM：奥法木单抗；Bz：硼替佐米；CAP：卡普塞珠单抗；CSA：环孢素；IVIG：静注人免疫球蛋白；Dara：达雷妥尤单抗；MMF：霉酚酸酯；CR：完全缓解；PR：部分缓解；NR：无效

CD38被认为是治疗浆细胞疾病和某些自身免疫性疾病的靶点，此前达雷妥尤单抗（人源化抗CD38单克隆抗体）已广泛用于多发性骨髓瘤的治疗[29]，近年来，陆续有学者将达雷妥尤单抗应用于治疗难治/复发的iTTP。van den Berg等[22]首次报道了达雷妥尤单抗治疗复发/难治的iTTP病例。2例患者在达雷妥尤单抗治疗后的两周内，抗ADAMTS13 IgG抗体滴度迅速降至正常，ADAMTS13活性也恢复正常。最近Weisinger等[23]发现，达雷妥尤单抗似乎在其他治疗策略（包括靶向T细胞的药物如环孢素A，甚至其他针对浆细胞的疗法如硼替佐米）失败的iTTP患者中仍然有效。未来在对一线治疗耐药或复发的iTTP患者中，针对浆细胞的治疗或许能发挥一定作用。然而，尚缺乏前瞻性数据来准确说明其安全性和疗效，亦无数据支持将针对浆细胞的治疗作为iTTP的首选方案。

FIB在血浆中浓度为2～4 g/L，低于2 g/L即可诊断为低纤维蛋白原血症。FIB减低的原因包括遗传性和获得性因素，而获得性FIB降低常见原因包括肝脏疾病、恶性肿瘤、浆细胞疾病、SLE、类风湿性关节炎、噬血细胞综合征、弥散性血管内凝血、药物等[30]。本例患者在治疗过程中反复出现纤维蛋白原减少，但她既往无低纤维蛋白原血症病史和家族史，基本排除了遗传性纤维蛋白原缺陷症。此外，该患者补充外源性纤维蛋白原后纤维蛋白原水平可回升至正常，基本排除了存在纤维蛋白原抗体的可能。Korkmaz等[31]报道了SLE合并纤维蛋白原减少的病例，他们推测抗DNA抗体加速了纤维蛋白原的代谢和消耗，故而随着SLE病情得到控制，纤维蛋白原可恢复至正常范围，这与本例患者情况相似。狼疮活动导致纤维蛋白原减少虽然少见，但在合并血小板减少时，可能会加重患者临床出血症状，应引起临床的重视。

iTTP作为临床急危重症，需要及时甄别，并启动包括TPE、激素、利妥昔单抗等在内的治疗。对于复发、难治的病例可尝试如硼替佐米等针对浆细胞的治疗。诊断iTTP的同时还需完善全面的检查，以排除合并其他自身免疫性疾病的可能。TTP本身可导致多系统损害，同时还可能合并其他多种免疫性疾病，组建MDT团队对于危重、复杂病例的诊治，制定个性化诊疗方案，及时挽救患者生命至关重要。
